# Linguistic Grammar Learning and *DRD2*-TAQ-IA Polymorphism

**DOI:** 10.1371/journal.pone.0064983

**Published:** 2013-05-31

**Authors:** Patrick C. M. Wong, Marc Ettlinger, Jing Zheng

**Affiliations:** 1 Department of Linguistics and Modern Languages, The Chinese University of Hong Kong, Shatin, Hong Kong SAR, PR China; 2 The Roxelyn and Richard Pepper Department of Communication Sciences & Disorders, School of Communication, Northwestern University, Evanston, Illinois, United States of America; 3 Department of Otolaryngology - Head and Neck Surgery, Feinberg School of Medicine, Northwestern University, Chicago, Illinois, United States of America; 4 Hugh Knowles Center for Clinical and Basic Science in Hearing and Its Disorders, Northwestern University, Evanston, Illinois, United States of America; Wake Forest School of Medicine, United States of America

## Abstract

As research into the neurobiology of language has focused primarily on the systems level, fewer studies have examined the link between molecular genetics and normal variations in language functions. Because the ability to learn a language varies in adults and our genetic codes also vary, research linking the two provides a unique window into the molecular neurobiology of language. We consider a candidate association between the dopamine receptor D2 gene (*DRD2*) and linguistic grammar learning. *DRD2*-TAQ-IA polymorphism (rs1800497) is associated with dopamine receptor D2 distribution and dopamine impact in the human striatum, such that A1 allele carriers show reduction in D2 receptor binding relative to carriers who are homozygous for the A2 allele. The individual differences in grammatical rule learning that are particularly prevalent in adulthood are also associated with striatal function and its role in domain-general procedural memory. Therefore, we reasoned that procedurally-based grammar learning could be associated with *DRD2*-TAQ-IA polymorphism. Here, English-speaking adults learned artificial concatenative and analogical grammars, which have been respectively associated with procedural and declarative memory. Language learning capabilities were tested while learners’ neural hemodynamic responses were simultaneously measured by fMRI. Behavioral learning and brain activation data were subsequently compared with the learners’ *DRD2* (rs1800497) genotype. Learners who were homozygous for the A2 allele were better at concatenative (but not analogical) grammar learning and had higher striatal responses relative to those who have at least one A1 allele. These results provide preliminary evidence for the neurogenetic basis of normal variations in linguistic grammar learning and its link to domain-general functions.

## Introduction

It is well documented that adults have difficulty learning a foreign language. In particular, syntax and other rules that govern combinatorial relationships of linguistic elements are difficult to learn to native-like attainment levels [1], [2]. Research has been conducted to identify factors that may contribute to learning success. Factors such as cognition (e.g., working memory [3], auditory [4], neuroanatomy [5], [6], and musical experiences [7], [8]) have all been linked to aptitude for foreign languages. Although being regarded as an important contributing factor, the ways in which genetic factors may be linked to foreign language learning has yet to be investigated. Using a candidate gene approach, we examined the link between a dopamine receptor gene and success in acquiring phonological grammar and its neural basis.

**Figure 1:**
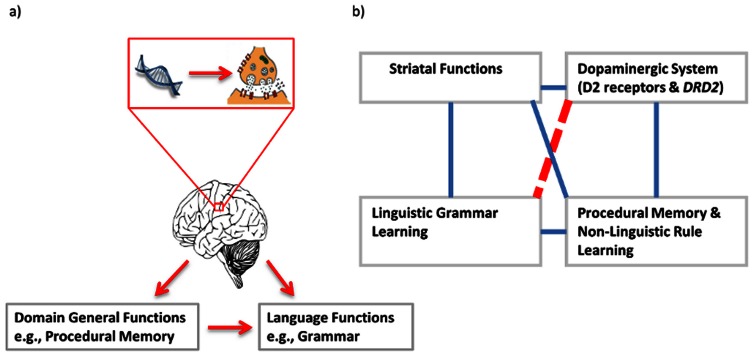
Relationships among neural systems, genes, general cognitive abilities, and language functions. At the systems-level, the brain controls linguistic and domain-general cognitive functions, which also influence linguistic functions; at the molecular-level (zoomed-in), genes influence the impact of neurotransmitters and neuronal processes (a). Solid lines in (b) represent known relationships found in the dopaminergic system; the dashed line represents the relationship being investigated (see main text for references for known relationships).

Several relationships among genes, neural systems, language, and cognition have been established that can form the basis for developing informed, genetic language learning hypotheses. Specifically, as depicted in Figure 1, relationships have been established between: 1) grammar learning and the fronto-striatal system [9]; 2) grammar learning (including sound pattern learning) and procedural memory (including non-linguistic rule learning) [10], [11]; 3) procedural memory (non-linguistic rule learning) and the fronto-striatal system [12], [13]; 4) fronto-striatal pathway and dopaminergic system [14]; and 5) most importantly, it has recently been found that the dopamine receptor D2 gene (*DRD2*) TAQ-IA polymorphism (rs1800497) is associated with non-linguistic rule learning, including reversal learning [15] and learning from feedback [16], learning skills that can be broadly construed as procedural in nature [17]. In addition, in verbal behaviors, *DRD2* has been linked to receptive vocabulary ability [18], whose learning is also related to procedural word segmentation [19]. Individuals with an A1 allele of *DRD2*-TAQ-IA have been found to have up to 30% reduction of dopamine receptor D2 density [20], [21] and receptor binding [22] in all areas of the human striatum.

Capitalizing on these five sets of relationships reported in the literature (Figure 1), we designed a study examining *DRD2*-TAQ-IA polymorphism and the learning of grammar that governs sound patterns in an artificial language. Foreign sounds and sound patterns are difficult to learn, and language learning is characterized by substantial individual differences, even among healthy adults [10], [2], [23]. The extent to which domain-general mechanisms, such as memory and non-linguistic rule learning, are integral to sound pattern learning is subject to debate [10], [24], [25]. Consequently, the role that specific genes play in grammar learning has yet to be explored, especially genes that are linked to domain-general cognitive functions. Based on the five sets of relationships reported, we hypothesized that carriers of the *DRD2*-TAQ-IA1 allele would show poor learning of phonological grammar relative to their A2 carriers. More specifically, we only expect poorer learning for the components of grammar that are tied to the procedural memory and dopaminergic system.

**Figure 2:**
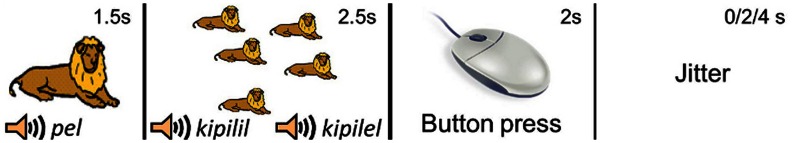
An example of a trial testing participants’ ability to apply the grammar they learned. Each trial is a version of a “wug”-test and participants performed this test in the scanner.

**Figure 3:**
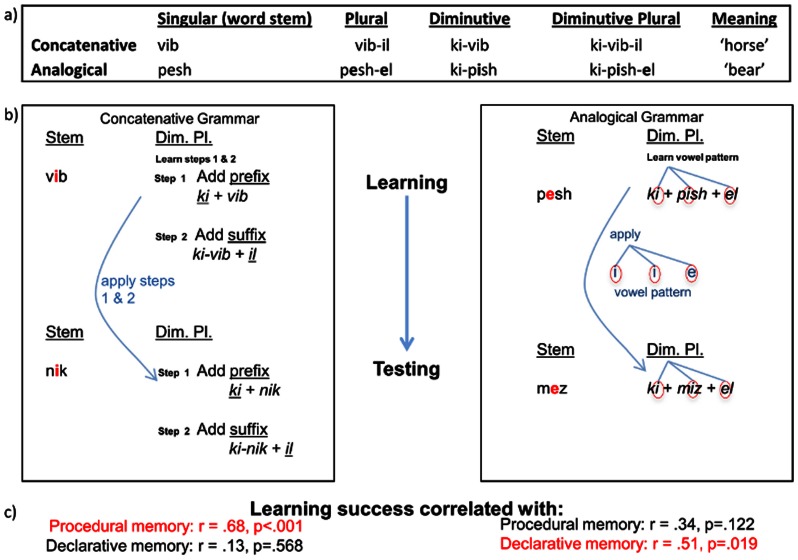
Concatenativeanalogical grammar: sample words (a), learning process (b), and correlation between learning and domain-general memory (c) are shown. Participants learned the two types of grammar in one language (/i/and/e/vowels triggered the application of the concatenative and analogical grammar, respectively) without any explicit instructions on how rules should be applied. The correlation between learning of these grammars and domain-general memory abilities provides further evidence of the underlying cognitive processes involved (c).

In this preliminary study, younger adult participants learned two types of grammars used to create new words as part of an artificial language modeled on Shimakonde, a Bantu language spoken in Mozambique [26]. Following training, they were tested on their ability to apply the learned grammars to untrained items while their cerebral hemodynamic responses were measured using fMRI (Figure 2). None of the participants were bilingual nor had any exposure either to Shimakonde or any language in the Bantu family. The *concatenative grammar* (see Figure 3), triggered by the/i/vowel in the word stem, consists of a process of concatenating the word stem (*vib* in the example given in Figure 3) with a suffix indicating the plural (*-il*) and/or prefix indicating the diminutive (*ki-*) to form derivatives (plural, diminutive and/or diminutive plural forms) of the word stem without changing any sounds in the word stem or affixes. The *analogical grammar,* triggered by the/e/vowel in the word stem, consists of a pattern transferal process, specifically in the form of an analogy of *trained singular stem* : *trained diminutive plural* :: *new singular stem* : *new diminutive plural* (e.g, p**e**sh : ki-pish-el :: m**e**z : ki-miz-el; the underlined i-i-e vowel pattern is analogized). Application of just the concatenation rule would result in the selection of the wrong vowel for/e/−stem words. Although different names have been used to describe these two types of grammars, their typological existence has been extensively documented in the literature [27]. In particular, it has been observed that the phonological realization of a morpheme in a concatenative grammar is determined exclusively by context (e.g. [28], whereas analogical grammar requires the use of analogizing to other word forms and therefore a different mechanism might be engaged (e.g. [28], [29], [30]). More specifically, previous research [10] and our current experiment (see below) confirmed that concatenative grammar learning is associated with the fronto-striatal system and procedural memory, while analogical grammar learning is at least partially dissociable from concatenative grammar learning and is associated with declarative memory. Analogical grammar learning serves as a control for the concatenative grammar learning wherein we expected the latter to be influenced by *DRD2*-TAQ-IA, but not the former. Given the similarity of the two grammars, this would suggest that A2 allele carriers of *DRD2*-TAQ-IA are not simply better at all experimental tasks or all language-learning tasks, but rather are better at a very specific aspect of language learning, namely the concatenative grammar.

## Methods

### Ethics Statement

This study was performed in strict accordance with an approved protocol. Participants provided informed written consent in accordance with the Institutional Review Board and all experimental procedures were approved by the Northwestern University Institutional Review Board.

### Participants

Participants were twenty-two native English-speaking adults [mean age = 22; 16 females] who reported having no audiologic and neurologic deficits. All subjects were right-handed as assessed by the Edinburgh Handedness Inventory [31].

### Language Learning

Participants were told that they would be exposed to word-picture pairings from a foreign language and that they would be tested afterwards on what they learned. Training was done in one session and involved passive exposure to the artificial language with the words played over headphones while a picture showing their meaning was projected on a screen. Learning was implicit with no feedback or explication of the rules provided.

Twelve (6 concatenative and 6 analogical) word stems were used in training and were presented in singular, plural (a picture of 4 items), diminutive (a picture one sixth the size of regular items) and diminutive plural (4 items, each one sixth the size) forms, resulting in a total of 48 words. The presentation order was blocked, with blocks for each form (singular, plural, diminutive, or diminutive plural) and rule type (concatenative or analogical), plus a rest block. These nine blocks were repeated four times in pseudo-random order such that the same block was not presented twice in succession. Within each block, the six words from the language for each rule type were presented twice so participants were exposed to each word eight times (2 repetitions in each block, each block repeated 4 times). Words were presented with a two-second SOA, so each block was 24 seconds long for a total training time of 15 minutes.

At the conclusion of training, participants were given a version of a “wug” test, which is used to assess knowledge of the grammar of a language, as opposed to the vocabulary [32]. This testing phase was performed inside the MRI scanner. Figure 2 shows an example of one trial in the testing phase. For each trial, participants were presented with one of 18 new words in the singular form and were prompted to select the correct form for a derived word using a button press from two alternatives. For example, the participants would see a picture of a chicken and hear the word *nik.* Then they would see a small chicken and hear *ki-nik* and *ki-nek*, and press the button corresponding to whether they thought the first or second alternative was the correct choice. For each trial, participants would see the first picture for 1.5 seconds and the second picture for 2.5 seconds while the two alternatives played over the headphones. The participant would then have 2 seconds to respond followed by either 0, 2 or 4 seconds of jitter as optimized by OptSeq [33] as the fMRI scanning protocal was rapid event related. There were 18 words (9/i/words, 9/e/words) tested for the 3 inflectional paradigms (plural, diminutive, and diminutive plural) in random order repeated 5 times, making a total of 270 trials over 36 minutes. Participants’ performance on these untrained items formed the measure of language learning success.

The training method used in the current study is adopted from our previous study [10]. Of note, Ettlinger et al. used several implicit and explicit training methods and the pattern of results in regard to successes and variability in the learning of the two grammars was very similar across the methods.

### fMRI Procedures

While participants performed the “wug” test for ascertaining their ability to apply the learned grammar to untrained items following training (as described above), hemodynamic responses were measured using fMRI. Magnetic resonance images were acquired using a Siemens 3T Trio MRI scanner. The T2*-weighted functional images were acquired axially using a susceptibility weighted EPI pulse sequence (TE = 20 ms, TR = 2000 ms, flip angle = 90°, in-plane resolution = 3.4375 mm×3.4375 mm, 38 slices with a slice thickness = 3 mm (without gap between slices) were acquired in an interleaved measurement). After the functional scan and behavioral task, an anatomical image was acquired axially using a high resolution, T1-weighted 3D volume (MP-RAGE; TR/TE = 2300 ms/3.36 ms, flip angle = 90°, TI = 900 ms, matrix size = 256×256, FOV of 22 cm, slice thickness = 1 mm). For analysis of the functional images, each individual’s signal was deconvolved using a general linear model using a 16^th^-order polynomial to model the baseline (because of the long time period). A 4-second BLOCK model was used to model the hemodynamic response functions for each condition starting at the onset of the presentation of the second option in the two alternative forced choice task. The resulting voxel-wise percent signal change was normalized for each participant. A mask for the striatum was determined for each individual participant by taking the region of interest map for the striatum (caudate, putamen and nucleus acumbens) for standard stereotaxic template (ICBM 452) and transforming it onto each participant’s T1-weighted anatomical image using a series of linear transformations as implemented in AFNI [34]. For all analyses, we corrected for multiple comparison using a Monte-Carlo simulation for a corrected *p*<.05. This corresponded to a statistical threshold for a single voxel of *p* = 0.00011 extending at least 370 mm^3^ in cluster size.

### Cognitive Testing

Participants’ declarative memory, working memory and procedural memory abilities were measured using the Visual-Auditory Learning and the Auditory Working Memory subtest of the Woodcock–Johnson III Tests of Cognitive Ability [35] and the Tower of London test (TOL) [36], respectively. Improvement in performance on the TOL over time is reflective of procedural learning [37]. The use of the TOL task serves to minimize the role of motor skills in performance as compared to other tests of procedural learning such as the serial reaction time task [38], thereby isolating higher-order procedural learning. For evaluating TOL performance, participants were evaluated based on time required to complete the repeated puzzles. A mean and standard deviation for the present participants was used to calculate a z-score for each participant representing their performance. Of the 22 participants tested, we were able to obtain working memory and declarative memory scores from 21 participants and procedural memory scores from all 22.

### Genomic Procedures

Participants’ *DRD2*-TAQ-IA (rs1800497) genotype was determined. DNA was extracted from buccal swab samples using QIAamp DNA mini-kit (Qiagen). Genotyping of *DRD2*-TAQ-IA was determined by polymerase chain reaction (PCR) amplification of DNA followed by TaqI enzyme digestion [39]. A1 genotype has a 310 bp-size band, while A2 has two bands: 130 bp and 180 bp. PCR conditions included denature at 94°C for 3 min followed by 45 cycles at 94°C for 30 sec, 55°C for 45 sec and finally, 72°C for 1 min (forward primer: 5′-CCGTCGACGGCTGGCCAAGTTGTCTA-3′; reverse primer: 5′-CCGTCGACCCTTCCTGAGTGTCATCA-3′). We expected those learners who are homozygous for A2 (A2/A2) (i.e., neither A1/A1 nor A1/A2 carriers) would be most successful in learning the concatenative grammar in which the contribution of the striatum is most needed.

## Results

### Language Learning and Memory

Consistent with our previous study [10], we found large individual differences in learning in both the concatenative [48% to 94% accuracy, mean = 71%, standard deviation = 14%] and analogical [33% to 88% accuracy, mean = 61%, standard deviation = 14%] grammar learning conditions. Also consistent with our previous study, learning success was significantly correlated with procedural memory in the concatenative [Pearson’s r = .684, *p*<.001] but not in the analogical grammar condition [Pearson’s r = .339, *p* = .122]. On the other hand, declarative memory was correlated with language learning in the analogical [Pearson’s r = .507, *p* = .019] but not the concatenative condition [Pearson’s r = .132, *p* = .568]. Working memory was not correlated with either the learning of analogical [Pearson’s r = .046, *p* = .842] or concatenative grammars [Pearson’s r = −.001, *p* = .962]. Age was not correlated with the learning of the analogical [Pearson’s r = .016, *p* = .942] or the concatenative grammar [Pearson’s r = .180, *p* = .423].

### DRD2-TAQ-IA and Language Learning

Participants were classified into two genotype groups–the A2/A2 group and the A1 group (comprised of both A1/A1 and A1/A2 carriers)–based on the presence of at least one A1 allele. The relationships among learning success, genotype, brain responses, working, declarative, and procedural memories were examined. Eight participants were classified into the A2/A2 group and 14 were classified into the A1 group. Only one of the 14 A1 subjects was homozygous for A1, which is consistent with the low frequency rate for A1/A1 carriers reported in the literature [40], [41]. Our sample was in Hardy-Weinberg equilibrium.

**Figure 4:**
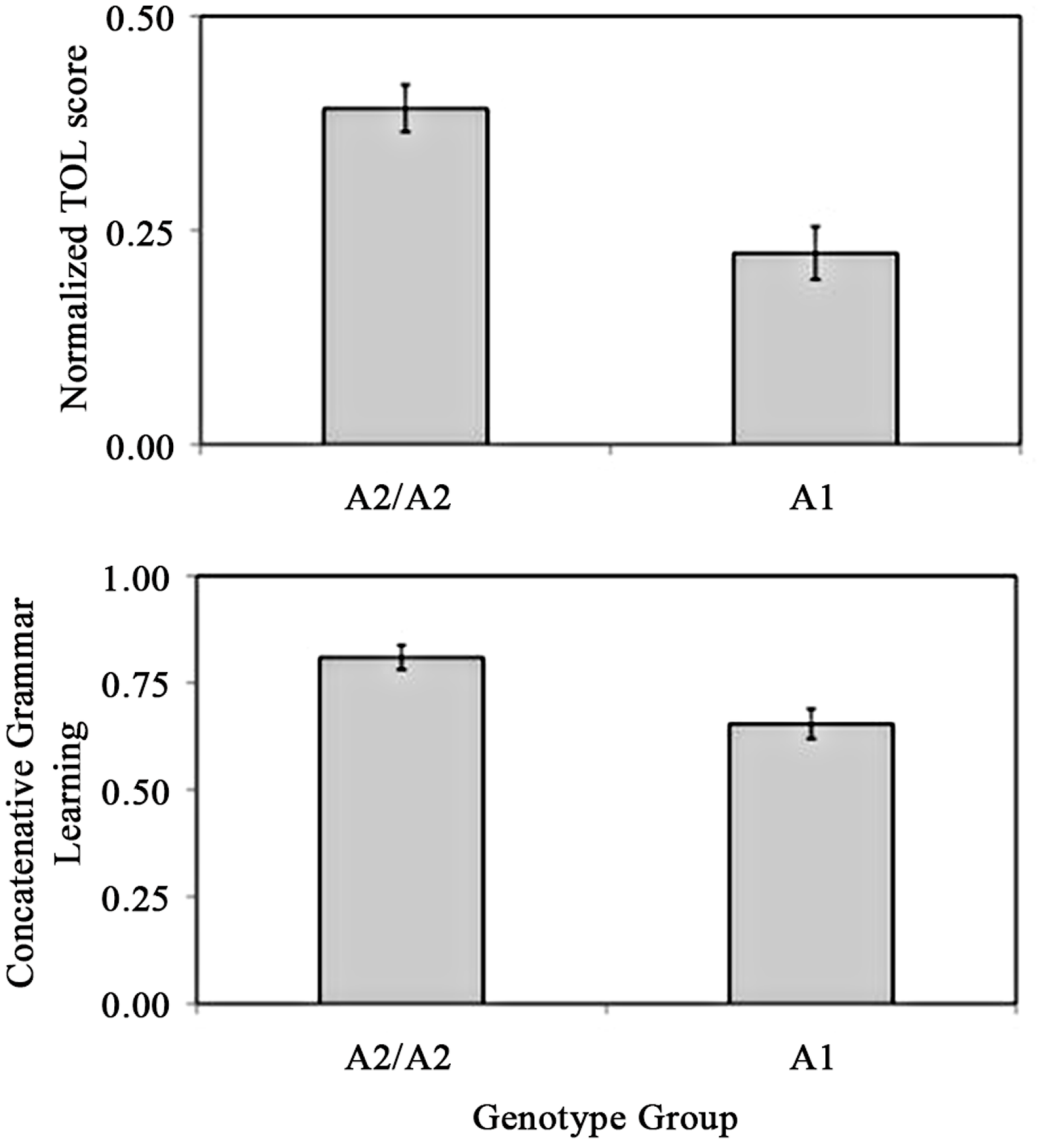
Performance on the Tower of London procedural memory test (TOL) (top) and language learning in the concatenative condition (proportion correct on untrained items) (bottom) by genotype groups (A2/A2 vs. A1). Error bars show standard error of the mean.

A one-way ANOVA revealed the A2/A2 group to have significantly better procedural memory (Figure 4, top panel) [F(1,20) = 13.373, *p = *.002, partial eta-squared = .401] but not working memory [F(1,19) = .482, *p = *.496, partial eta-squared = .025] nor declarative memory [F(1,19) = .696, *p = *.414, partial eta-squared = .035]. For our sample, the A2/A2 group [mean = 24.86] was significantly (and unexpectedly) older than the A1 group [mean = 20.93] [F(1,20) = 6.558, *p = *.019, partial eta-squared = .247]. We did not expect age to contribute to language learning differences because all participants were younger adults; nevertheless, subsequent analyses below incorporate age to be a co-variate in light of the observed group difference.

For the concatenative grammar condition, a one-way ANOVA revealed the A2/A2 group to have significantly better language learning (Figure 4, bottom panel) [F (1, 20) = 9.389, *p = *.006, partial eta-squared = .319], even after age and working memory were used as co-variates [F (1, 17) = 7.389, *p = *.015, partial eta-squared = .303]. However, when procedural memory was controlled, no significant group difference in concatenative grammar learning was found [F (1, 19) = 1.100, *p = *.307, partial eta-squared = .055], suggesting that non-linguistic procedural memory and concatenative grammar learning are likely mediated by the same domain-general cognitive ability [9], [10]. For analogical grammar, the two genotype groups did not differ in their language learning [F (1, 20) = .099, *p* = .757, partial eta-squared = .005].

### DRD2-TAQ-IA and fMRI

We also examined hemodynamic responses to untrained stimuli (in the form of a “wug” test) between the two genotype groups to provide evidence that the mechanism of the genetic influence on language-learning is mediated by the striatum, as we hypothesize. Table 1 summarizes the results for a whole-brain analysis of the concatenative and analogical grammar conditions.

**Table 1:** Voxel-wise comparisons between brain responses in the A2/A2 and A1 groups in the concatenative grammar (a) and analogical grammar conditions (b).

Activation peak	Talairach coordinates x,y,z		Cluster size (voxels)		Peak T value
***(a) Concatenative Grammar (A2/A2> A1)***					
Left putamen	−31, −7, −4		2042		5.1
Right putamen	31, 11, −3		1953		5.3
Right Brodmann area 6	42, 10, −58		1133		5.9
Cerebellum	17, −94, 18		635		6.4
Right inferior frontal gyrus (p. orbitalis)	49, 23, 3		531		5.9
Left inferior parietal lobule	−55, −37, −53		229		5.3
Left lentiform nucleus	−12, −6, 4		225		5.3
Left inferior frontal gyrus (p. orbitalis)	−49, 27, 3		212		5.5
***(b) Analogical Grammar (A2/A2> A1***					
Right Brodmann area 6		42, 10, 58		701	5.4
Right inferior frontal gyrus (p. orbitalis)		49, 23, −3		524	5.3
Left inferior frontal gyrus (p. orbitalis)		−49, 27, −3		435	6.0
Left insula		−33, −6, 9		399	6.0
Cerebelum		47, −72, −23		348	5.2
Right fusiform gyrus		18, −96, −17		276	5.8
Left putamen		−26, −8, 15		217	5.2
Left inferior parietal lobule		−55, −37, 54		205	6.0

Clusters that exceeded the statistical threshold for multiple comparisons are shown.

**Figure 5:**
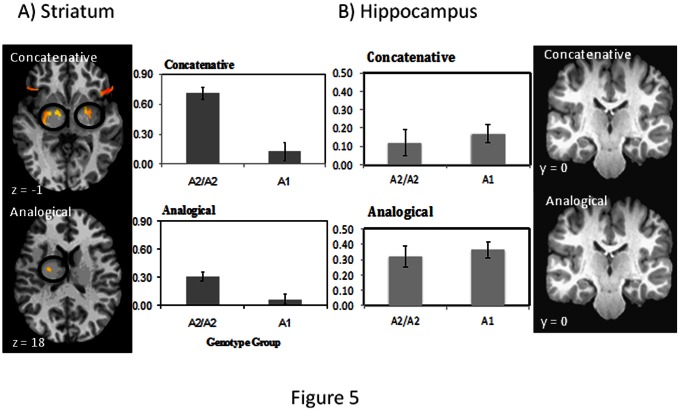
(A) Brain activation differences in the striatum between the A2/A2 and the A1 group in the concatenative grammar and analogical grammar learning conditions. Circled regions on the left panels highlight activation differences in the striatum (warmer color shows higher t-values, see Table 1); note that activation in the inferior frontal region can also be seen on the concatenative slide. Right half of (A) show averaged activities (% signal change) in the striatum bilaterally for each condition in each group. (B) Brain activation differences in the hippocampus between the A2/A2 and the A1 group in the concatenative grammar and analogical grammar learning conditions. No regions show any significant differences. Left half of (B) shows averaged activities (% signal change) in the hippocampus bilaterally for each condition.

Dopamine receptors have been found in the human brain in these regions, particularly the striatum [42]. These are also regions previously implicated in grammar learning [43] and various forms of non-linguistic procedural learning [12], [13]. Because our candidate gene is specifically implicated in striatal functions [22], we performed a 2 (genotype group) × 2 (grammar) repeated measures ANOVA specifically on this region defined anatomically. Figure 5 summarizes these key results regarding *DRD2* and brain responses in each condition. We found significant effects of grammar (concatenative vs. analogical) [F (1, 20) = 10.274, *p* = .004, partial eta-squared = .285] and group (A2/A2> A1) [F (1, 20) = 23.911, *p*<.001, partial eta-squared = .503], and a significant interaction effect [F (1, 20) = 5.730, *p* = .027, partial eta-squared = .151] with a larger group difference in the learning of concatenative grammar. In particular, in the concatenative grammar condition, significant group differences were found in the striatum (especially in the putamen) and inferior frontal regions bilaterally, with the A2/A2 group showing stronger responses than the A1 group (post-hoc t-test, (*t* (21) = 4.0, *p*<.001; Cohen’s d = 1.97; Figure 5A). To a much smaller extent, group differences were also found in these dopamine-related regions in the analogical grammar condition as indicated by the interaction effect in the ANOVA. By comparison, there was no evidence for a difference between the two group using the hippocampus as the region of interest [interaction effect: *F* (1, 20) = .60, *p* = .87) (Figure 5B)].

## Discussion

Although previous research has examined the relationships between genes and language impairments and language delays (for work on communicative impairments and developmental delay, see [44], [45], [46] for genes *ROBO1*, *FOXP2*, and *CNTNAP2*, respectively), it has yet to examine normal variation in language learning. As far as we are aware, our study is the first to demonstrate the neurogenetic basis of aspects of such variability in learning. Our results point to the specific contribution of a gene that potentially influences the impact of dopamine. Our results support the hypothesis that concatenative grammar learning is associated with the fronto-striatal system and procedural memory, corroborating previous research on the link between procedural memory and grammar [11], [19], [47], including for the specific grammatical pattern that subjects learned [10].

The lack of relationship between *DRD2*-TAQ-IA and the analogical grammatical pattern serves as a control, suggesting that *DRD2*-TAQ-IA is not simply supporting better language learning and general stimulus-response learning. Though there has been debate as to the specific cognitive mechanisms involved in the analogical grammatical pattern used in this study [28], the/e/−stem pattern is acknowledged as more complex than simple concatenation [28], [48] and is argued to be processed via analogy or some other mechanism distinct from the basic concatenation used in the concatenative condition [29]. Previous research has confirmed that learning analogical patterns is at least partially dissociable from concatenative grammar learning and is associated with declarative memory [47], including for the specific analogical pattern subjects learned in the present study. This analogical pattern may be considered similar to the irregular past tense in English and the concatenative condition being similar to the regular past tense [49] (Figure 3). Although *DRD2* has been linked to psychiatric conditions [42] and non-linguistic rule learning [16], a specific relationship with linguistic learning has not been previously documented, as far as we are aware.

It is important to note that although much evidence points to *DRD2*-TAQ-IA being associated with *dopamine* receptor D2 binding/expressing density [21], the TAQ-I cut site is located within exon 8 of the adjacent gene: ankyrin repeat and protein kinase domain-containing protein 1 (*ANKK1*) on chromosome 11 [50]. Thus, it could be difficult to disentangle the direct contribution of *DRD2*-TAQ-IA or nearby genes such as *TTC12* (tetratricopeptide repeat protein 12) and *ANKK1* [51], [52], [53], [54]. In addition to the *DRD2*-TAQ-IA site we investigated, other SNPs around the *DRD2* gene, such as rs12364283, rs228265, rs1076560, and rs6277, may also influence *DRD2* functions [55], [56]. These various SNPs influence quantity of *DRD2* mRNA, different isoforms, as well as presynaptic relative to postsynaptic dopamine receptors D2 in the brain. Whether or not these SNPs can directly influence language learning/procedural learning system require further investigations.

It is worth noting that the genetic polymorphism we examined has been investigated in other learning studies (though not specifically in language learning), which included a similarly smaller sample size and used a hypothesis-driven, candidate gene approach [15], [16]. Although our sample size is relatively small (albeit with significant results), the current study is at least the third that points to the importance of *DRD2*-TAQ-IA in learning, and the contribution of *DRD2*-TAQ-IA to learning is unlikely to be spurious. Furthermore, our study does not rely only on evidence from one modality, but rather gene, brain, and cognitive measures were assessed and provided converging evidence for the relationship between dopamine and grammar with brain and cognitive systems as mediating factors. Moreover, by demonstrating a distinction between the underlying factors (cognitive, neural, and genetic) that modulate two different types of grammar learning, we can observe a relationship between *DRD2*-TAQ-IA and the acquisition of a specific component of language (concatenative simple grammar in this case), rather than demonstrating the general effects of varying abilities in overall language learning, intelligence, or test taking. Although consistent with some similar published work, we acknowledge that our sample size is small and therefore future replication studies should be conducted with a larger sample size. The current study should only be viewed as a preliminary study that has demonstrated a link between genetics and language learning.

As the present study provides evidence for a domain-general molecular and cognitive basis for one type of grammar learning, it provides support for theories that argue against a domain-specific view of language learning [24]. Consequently, the present study would also speak to a hypothesis that links subtle variations in other procedurally-based non-linguistic skills and some specific types of grammar learning. Future research is required to establish such relationships.

Results from the present study generate a number of questions to be examined by future studies. For example, it is not known whether genetic differences (*DRD2* and other genes) may contribute to normal variation in first language acquisition [57] and cross-cultural linguistic typological differences [58]. It is also not known whether *DRD2*-TAQ-IA contributes to communicative impairments and developmental delay, and whether it contributes to the interaction between vocabulary [18] and grammar learning. Furthermore, the variability in language learning within genotype groups calls for investigations on environmental factors, other genetics factors, and their interactions that may lead to differences in success. It is especially worth noting that a recent study has reported that teacher quality can moderate genetic effects on early reading [59], suggesting that genetic biases can interact with how training is provided. As natural language learning can take very different forms than the paradigm used here, as with classroom-based and immersion-based learning [23], methods of training and their interactions with genetic predispositions should be examined in future research. It should be noted that as language instruction becomes more automated and computerized [60], [61], understanding language learning in controlled settings, as in this experiment, becomes increasingly valuable.
